# A Combination of Tachycardia-Mediated Heart Failure and Coronary Artery Vasospasm-Induced Silent Myocardial Infarction in a Patient with Severe Thyrotoxicosis

**DOI:** 10.1155/2018/4827907

**Published:** 2018-03-11

**Authors:** Serena Sert Kim Khoo, Chong Mow Chu, Yin Khet Fung

**Affiliations:** ^1^Endocrinology Unit, Queen Elizabeth Hospital II, Kota Kinabalu, Sabah, Malaysia; ^2^Cardiology Department, Queen Elizabeth Hospital II, Kota Kinabalu, Sabah, Malaysia

## Abstract

Severe thyrotoxicosis can present with a myriad of cardiovascular complications. It may be mild features such as palpitations, tachycardia, and exertional dyspnea or may progress to life-threatening consequences such as atrial fibrillation, tachyarrhythmias, heart failure, myocardial infarction, and shock. In rare cases, they may present with myocardial ischemia secondary to coronary artery vasospasm. We report a case of a 59-year-old Malay gentleman who presented with fast atrial fibrillation and tachycardia-mediated heart failure that evolved to a silent myocardial infarction secondary to severe coronary artery vasospasm with undiagnosed severe thyrotoxicosis. He had complete resolution of heart failure and no further recurrence of coronary artery vasospasm once treatment for thyrotoxicosis was initiated and euthyroidism achieved. This life-threatening consequence has an excellent prognosis if recognised early and treated promptly.

## 1. Introduction

The cardiovascular manifestations from hyperthyroidism are well described and may include palpitations, tachycardia, exercise intolerance, dyspnea on exertion, widened pulse pressure, and atrial fibrillation that can evolve to tachyarrhythmias, heart failure, cardiovascular collapse, and shock in the acute setting of thyroid storm [1]. In rare instances, patients with hyperthyroidism can experience angina suggestive of myocardial ischemia either due to known or suspected underlying ischemic heart disease or secondary to coronary artery vasospasm (CAS) [2].

We report the case of a patient with severe thyrotoxicosis who presented with a spectrum of cardiovascular complications of fast atrial fibrillation and congestive cardiac failure who developed silent acute myocardial infarction as a consequence of severe diffuse coronary artery vasospasm.

## 2. Case Report

A 59-year-old previously healthy Malay gentleman presented with progressive worsening dyspnea, orthopnea, and paroxysmal nocturnal dyspnea for 1 month. He had no angina, palpitations, or ankle oedema. He had a 20-pack-year smoking history but had no other cardiovascular risk factors. He also had epigastric discomfort and vomiting.

Physical examination revealed a thin, restless but oriented man. His vital signs were as follows: blood pressure of 130/87 mmHg, irregular pulse rate of 150 beats/min, respiratory rate of 32/min, and temperature of 37.3°C. Cardiovascular examination revealed a raised jugular venous pressure, displaced apex beat, bibasal crepitations, and minimal ankle oedema. His baseline laboratory findings on admission are listed in Table 1.

**Figure 1:**
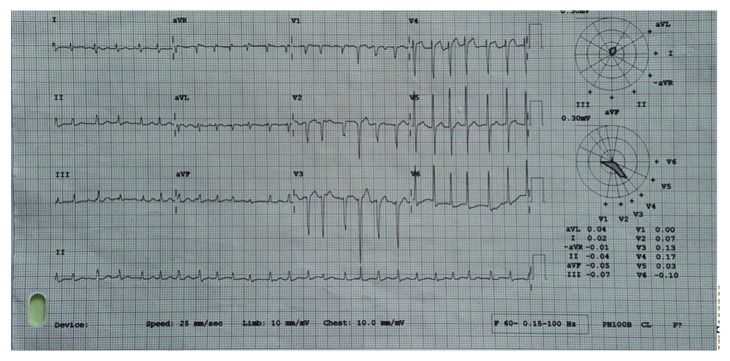
ECG on admission showing fast atrial fibrillation.

ECG on presentation showed fast atrial fibrillation with a ventricular rate of 156 beats per minute (Figure 1). Chest X-ray revealed signs of pulmonary congestion and cardiomegaly. Echocardiography showed mild to moderate left ventricular dysfunction with left ventricular ejection function of 40–50% with moderate mitral regurgitation, severe tricuspid regurgitation, and impaired right ventricular function. The mean estimated pulmonary artery pressure is 31 mmHg. No diastolic study was done in view of atrial fibrillation.

**Figure 2:**
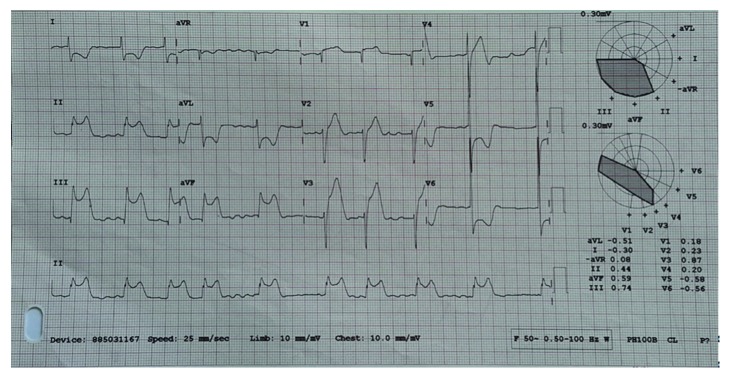
An immediate 12-lead ECG recorded atrial fibrillation with bradycardia and ST elevation in leads II, III, avF, and V1–V4 with ST depression in I, aVL, and V5-V6.

He was admitted and stabilised in the CCU and commenced on intravenous frusemide infusion, spironolactone 12.5 mg daily, carvedilol 9.375 mg twice daily, perindopril 2 mg daily, and thiamine and anticoagulated with s/c Clexane. The next day, he was noted to have fluctuating ST segment elevation on the cardiac monitor but was asymptomatic. An immediate ECG revealed marked ST segment elevation in leads II, III, aVF, and V1–V4 with ST depression in I, aVL, and V5-V6 with atrial fibrillation and bradycardia (Figure 2). Minutes later, he developed cardiogenic shock with a blood pressure of 68/43 mmHg and bradycardia.

**Figure 3:**
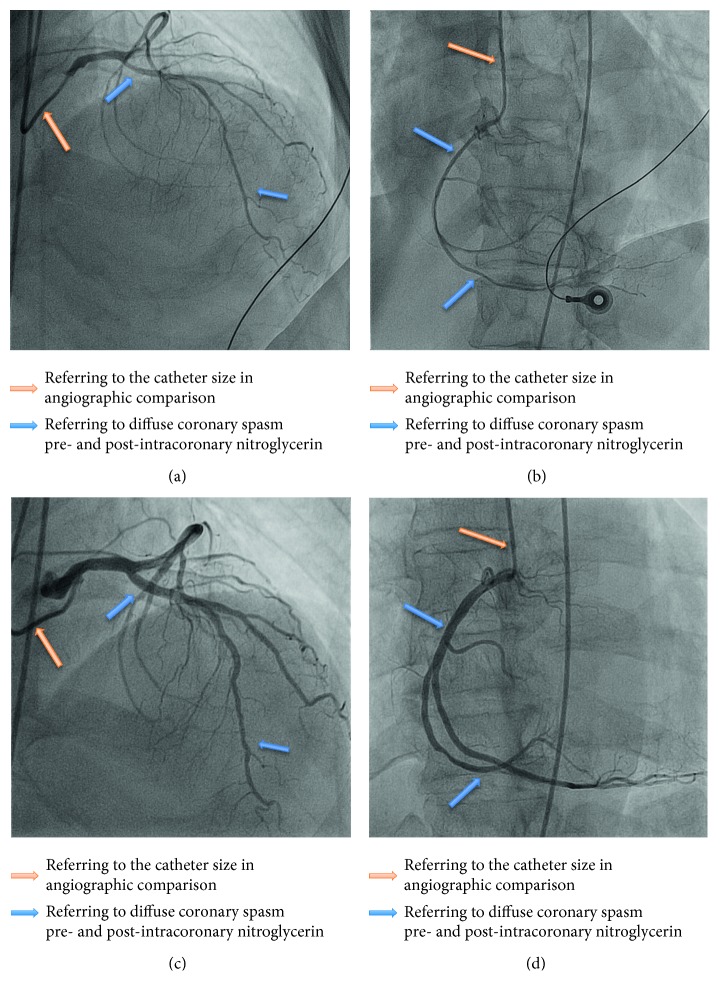
(a) Left coronary artery: diffuse coronary spasm involving proximal to distal of left anterior descending artery. (b) Right coronary artery: diffuse coronary spasm involving proximal to distal of right coronary artery, posterior descending artery, and posterior left ventricular branch. (c) Left coronary artery vasospasm resolved after intracoronary nitroglycerin. (d) Right coronary artery vasospasm resolved after intracoronary artery nitroglycerin.

An immediate coronary angiography was performed which revealed diffuse coronary artery vasospasm. Diffuse small caliber vessels were demonstrated in the left coronary artery (Figure 3) and the right coronary artery (Figure 3). Following intracoronary injection of 200 mcg nitroglycerin, the caliber of the vessel improved significantly as visualized in Figure 3 for the left coronary artery and Figure 3 for the right coronary artery. There was improvement in myocardial brushing, and no coronary artery stenosis was seen. He was commenced on continuous nitroglycerin infusion, and ST elevation reduced remarkably after coronary angiogram.

He developed transient episodes of bradycardia after procedure and required IV atropine and IV adrenaline and brief cardiopulmonary resuscitation. The patient was treated with IV infusion of nitroglycerin and titrated according to the ECG and clinical symptoms.

Later, on further questioning, he had thyrotoxic symptoms of heat intolerance, sweating, weight loss, and muscle weakness for several months. He had not noticed any neck swelling, obstructive symptoms, or visual symptoms. On reexamination, he had fine tremors, lid retraction, lid lag, and bilateral mild proptosis to suggest Graves' ophthalmopathy. There was a grade II diffuse goiter with no thyroid bruit. His Burch-Wartofsky score was 65 (>45) (Table 2).

Thyroid function test results were as follows: free T_4_ 61.6 pmol/L (normal range: 9–25), free T_3_ 21.8 pmol/L (normal range: 3.5–6.5), and TSH 0.001 uIU/ml (normal range: 0.4–4.7). There was no TSH receptor antibody level.

He was commenced on IV hydrocortisone, carbimazole, oral Lugol's iodine, propranolol, Cardiprin, warfarin, and oral mononitrates. He improved remarkably with complete resolution of hyperthyroid and heart failure symptoms 3 weeks after. He reverted back to sinus rhythm, and his repeat echocardiogram showed marked improvement of his left ventricular and right ventricular function, LVEF 60–65%, and pulmonary pressure not raised demonstrated by no significant TR and PR. His thyroid function test 2 months after improved with FT_4_ 15.21 pmol/L (9–19) and TSH < 0.01 uIU/ml, and he was scheduled for a radioactive iodine therapy.

## 3. Discussion

This report highlights a spectrum of cardiac complications in a patient with delayed diagnosis of severe thyrotoxicosis presenting with fast atrial fibrillation and heart failure evolving into a painless acute myocardial infarction secondary to diffuse severe coronary vasospasm.

Thyrotoxicosis affects the cardiovascular system via T_3_-mediated effects by increasing cardiac contractility, enhancing systolic and diastolic function, decreasing systemic vascular resistance and increasing circulating blood volume leading to increased preload, decreased afterload, and therefore increasing cardiac output by up to 300% from a euthyroid state [3].

This patient presented with classical tachycardia-mediated heart failure and fast atrial fibrillation. This mechanism led to an increased level of cytosolic calcium during diastole with reduced ventricular contractility and diastolic dysfunction. Increase in pulmonary artery pressure as in this case is contributory and a recognised cause of isolated right heart failure. In addition, 5% to 15% of thyrotoxic patients with atrial fibrillation are most likely to develop heart failure [3]. Beta-blockers can provide immediate relief of heart failure symptoms by slowing the heart rate and improving left ventricular function; however, cautious use in patients with true heart failure is advised. Marked symptoms resolution is seen upon attaining euthyroidism [4].

This case was further complicated with the development of painless acute myocardial infarction secondary to diffuse severe coronary vasospasm. The prevalence rate of coronary artery vasospasm with thyrotoxicosis is reported about 7.4% occurring more frequently in females, with age ranging between 44 and 75 years, and in Asians [5]. Smoking is a major risk factor for coronary artery vasospasm, and in this case it may be contributory [6].

Patients with coronary vasospasm with thyrotoxicosis typically present with either resting or exertional angina or syncope. However, it is important to note that the incidence of silent myocardial ischemia secondary to coronary vasospasm is more than twice than that of symptomatic ischemia [7] as in this case where the phenomenon was detected incidentally from ECG or cardiac monitoring.

Lee et al. described that coronary vasospasm with thyrotoxicosis presented with a higher incidence of acute myocardial infarction and angiographically had normal coronary arteries and were more spontaneous, diffuse, involving the left main vessel, and medically intractable compared to those without thyrotoxicosis [8]. This patient's presentation and angiographic findings were spontaneous, diffuse, and involving both coronary arteries and responded remarkably to intracoronary nitroglycerin.

The mechanism for the intensive spasm is still unclear. However, Napoli et al. demonstrated that vascular endothelium is a specific target of thyroid hormone evidenced by marked basal vasodilation contributed by excessive endothelial nitric oxide production coupled by exaggerated vascular reactivity because of enhanced sensitivity of the endothelial component and increased vasoconstrictory response to norepinephrine during the hyperthyroid state. This abnormal vasoconstrictory response reverses when euthyroidism is achieved [9]. This phenomenon not only involves the coronary arteries but also reported cerebrovascular occlusive disease (Moyamoya phenomenon) in uncontrolled Graves' disease that may share the same mechanism for acute vasospasm of the cerebral arteries [2].

Even though the clinical presentation of tachycardia-mediated heart failure along with CAS with thyrotoxicosis was severe, the clinical course became stable with excellent prognosis once the patient achieved euthyroidism [10, 11]. An important lesson to learn is the prompt clinical recognition and diagnosis of thyrotoxicosis as a cause of heart failure and coronary vasospasm. In this case, once the diagnosis of severe thyrotoxicosis was made and treatment initiated, the patient gradually improved with resolution of heart failure and hyperthyroid symptoms, reversion back to sinus rhythm, and improvement in echocardiogram findings and thyroid function levels.

## 4. Conclusion

Severe thyrotoxicosis can manifest with a myriad of cardiac complications. Tachycardia-mediated heart failure along with atrial fibrillation and coronary artery vasospasm is a life-threatening consequence of thyrotoxicosis, and the delay in diagnosis and treatment of hyperthyroidism in these patients may result in complications and unnecessary interventions. However, if recognised and treated early, it can lead to excellent prognosis.

## Figures and Tables

**Table 1:** Laboratory findings on admission.

	Result	Normal
FBC		
Hb	12.9 g/dl	13–17
Platelet	264	150–410
WBC	12.9	4–10

Renal		
Na^+^	141 mmol/L	136–145
K^+^	4.9 mmol/L	3.5–5.1
Urea	8.5 mmol/L	3–9.2
Creatinine	70 *µ*mol/L	63.6–110.5

Liver function		
Total protein	73 g/L	64–83
Albumin	29 g/L	38–54
ALT	256 U/L	0–55
ALP	142 U/L	40–150
AST	269 U/L	5–34

Coagulation		
PT	18.9 s	12.2–14.3
PTT	43 s	34.1–45.8

Cardiac		
hs-Trop	74.4 pg/mL	<34.2
CK	26 U/L	30–200
LDH	230 U/L	125–220

**Table 2:** Burch-Wartofsky score.

Temperature	5
GI dysfunction	10
Central nervous system	0
Tachycardia	25
Congestive cardiac failure	10
Atrial fibrillation	10
History	0
Total	65
